# The Turkish validity and reliability study of the Chronic Conditions Physician–Patient Relationship Scale in family medicine practice

**DOI:** 10.1017/S1463423626101236

**Published:** 2026-05-15

**Authors:** Duygu Ayhan Başer, Ogulcan Çöme, Emre Sari, Nezaket Sabir

**Affiliations:** 1 Department of Family Medicine, Hacettepe University Faculty of Medicine, Ankara, Türkiye; 2 Department of Family Medicine, Dokuz Eylul University Faculty of Medicine, Türkiye; 3 Department of Family Medicine, Cumhuriyet University Faculty of Medicine, Türkiye

**Keywords:** chronic diseases, family medicine, physician–patient relationship, primary care, reliability, validity

## Abstract

**Objective:**

This study aimed to adapt the Chronic Conditions Physician–Patient Relationship Scale (CC-PPR) into Turkish and to examine its validity and reliability among patients with chronic diseases receiving care from family physicians.

**Methods:**

A methodological study was conducted with 254 adult patients attending the Family Medicine Centers between May 01-October 01, 2025. The adaptation process followed World Health Organization guidelines. Construct validity was examined using confirmatory factor analysis (CFA), and reliability was assessed through internal consistency (Cronbach’s α, McDonald’s *ω*) and item–total correlations.

**Results:**

The CFA supported the original one-factor, 22-item structure with an excellent model fit (*χ* ^2^[209] = 59.847, *p* = 1.000; comparative fit index [CFI] = 1.000; Tucker–Lewis index [TLI] = 1.016; root mean square error of approximation [RMSEA] = 0.000; standardized root mean square residual [SRMR] = 0.048). Sampling adequacy was good (Kaiser–Meyer–Olkin [KMO] = 0.970; Bartlett’s *χ* ^2^[231] = 5934.429, *p* < 0.001). All standardized factor loadings were high (0.63–0.81, *p* < 0.001). Internal consistency was excellent (Cronbach’s *α* = 0.977; McDonald’s *ω* = 0.976), and corrected item–total correlations ranged from 0.74 to 0.86. Marital status, employment status, and type of health institution were significantly associated with relationship scores (*p* < 0.05).

**Conclusion:**

The Turkish version of the CC-PPR is a psychometrically robust, unidimensional, and reliable tool for evaluating the quality of family physician–patient relationships among individuals with chronic conditions. It can be used to assess communication and relational competencies of family physicians, support patient-centred care initiatives in chronic disease management.

## Introduction

Chronic diseases represent a major global health concern, leading to reduced quality of life, long-term treatment needs, and increased healthcare costs (Bilgiç and Pehlivan, 2023). Effective management of these conditions fundamentally depends on a strong and trust-based doctor–patient relationship, which influences treatment adherence, clinical outcomes, and overall patient satisfaction.

Most of the currently available doctor–patient relationship scales have been developed from psychotherapeutic frameworks and therefore fail to adequately assess the expectations and experiences of patients with medical conditions (Van der Feltz-Cornelis, 2004; Kaba and Sooriakumaran, 2007; Arbuthnott and Sharpe, 2009; Van Hoof, 2009; Kenny *et al.*, 2010; Eveleigh, 2012; Kurlander *et al.*, 2017). Moreover, the number of instruments specifically designed for individuals with chronic diseases remains limited. Hence, there is a clear need for a reliable, valid, and patient-centred tool that captures observable physician behaviours and can be applied in the context of chronic disease management (Gültekin, 2016).

The management of chronic illnesses requires not only medical expertise but also sustained collaboration between physicians and patients (Fuertes *et al.*, 2007; Sheridan *et al.*, 2015; Street, 2019). A robust therapeutic alliance facilitates medication adherence, lifestyle modification, and continuity of care. However, existing measurement tools and conceptualizations of the doctor–patient relationship are largely based on researchers’ or physicians’ perspectives, with insufficient incorporation of patients’ views. To address this gap, the *Chronic Conditions Patient–Physician Relationship Scale (CC-PPR)* was developed by Eigeland and Jones (2025) as the first patient-derived instrument specifically designed to evaluate how individuals with chronic conditions perceive their relationship with their physicians (Eigeland *et al*., 2025).

In primary care, family physicians play a pivotal role in the long-term management of patients with chronic diseases. Their continuous contact with patients provides a unique opportunity to establish trust, enhance adherence, and deliver holistic, patient-centred care. Nevertheless, most existing doctor–patient relationship scales are rooted in short-term, specialty-based clinical encounters or psychotherapy settings, and therefore do not sufficiently reflect the longitudinal, comprehensive, and relational nature of primary care practice (Pomey, 2015).

Consequently, adapting and validating the CC-PPR scale for the Turkish context is of great importance. Such adaptation will enable family physicians to assess and strengthen the quality of their relationships with chronically ill patients and contribute to the promotion of patient-centred care in primary health settings. Furthermore, a validated Turkish version will serve as a valuable research and clinical tool for measuring relational quality, improving patient experiences, and enhancing continuity and coordination of care within the framework of chronic disease management.

The aim of the present study is to adapt the *Chronic Conditions Patient–Physician Relationship Scale (CC-PPR)* into Turkish and to evaluate its psychometric properties in terms of validity and reliability. Through this adaptation, it will be possible to more accurately and comprehensively assess the quality of the relationships that family physicians establish with patients living with chronic diseases in Türkiye, thereby contributing to the improvement of patient–physician communication and chronic disease management within primary care settings.

## Methods

### Study setting and period

The methodological study was conducted at the Family Medicine Outpatient Clinics of Hacettepe University, Faculty of Medicine. The research began following approval from the institutional ethics committee and was completed between May 01, 2025, and October 01, 2025.

### Study population and sample

The study population consisted of individuals aged 18 years and older who applied to the Hacettepe University Family Medicine Outpatient Clinics for any reason. The CC-PPR contains 22 items (Eigeland *et al*., 2025). According to methodological recommendations that the sample size should be at least 10 times the number of items, a minimum of 220 participants was targeted (Akgül and Çevik, 2003).

#### Inclusion criteria

Being 18 years of age or older

Having a diagnosis of a chronic disease for at least 3 months

Voluntary participation

Having visited a family health centre after the diagnosis of a chronic disease

#### Exclusion criteria

Presence of orientation or cooperation problems

### Instruments and data collection

The study assessed the Turkish validity and reliability of the Chronic Condition Physician–Patient Relationship Scale (CC-PPR) (Annex-5) (Eigeland *et al*., 2025). Permission for translation and adaptation was obtained via e-mail from the original author, Dr. Jessica A. Eigeland (Annex-4). The original CC-PPR was developed through a three-round Delphi process conducted with Australian participants living with chronic physical health conditions, to define what constitutes a good doctor–patient relationship. The process resulted in a single-factor, 22-item scale demonstrating excellent internal consistency (Cronbach’s *α* = 0.97). Participants rated how often physicians displayed certain behaviours using a 7-point Likert scale ranging from 1 (never) to 7 (always). Higher scores indicated a stronger perceived doctor–patient relationship (Eigeland *et al*., 2025).

In this study, participants completed a sociodemographic questionnaire along with the Turkish version of the CC-PPR (Annex-3).

### Cross-cultural adaptation process

The cross-cultural adaptation followed the World Health Organization (WHO) guidelines and relevant methodological literatüre (Organization, 2016). The steps were as follows:

- **Forward Translation:**

Two independent native Turkish speakers translated the scale from English into Turkish – one a professional translator unfamiliar with the topic, and the other a family physician informed about the study objectives. The translations were compared, and discrepancies were resolved to create a single reconciled version.

- **Expert Panel Review:**

Ten experts in family medicine and health sciences reviewed the original, translated, and previously adapted versions of the scale. Each item was rated on a 4-point relevance scale (1 = not appropriate, 4 = highly appropriate). Based on expert feedback, the content validity index (CVI) was calculated. The content validity of the Turkish version of CC-PPR was evaluated by ten experts. As each item was rated on a 4-point relevance scale. The I-CVI values ranged from 0.90 to 1.00. The Scale-level CVI based on the average method was 0.9

- **Back Translation:**

The reconciled Turkish version was translated back into English by an independent bilingual translator unaware of the study and the original scale. The back-translated version was sent to Dr. Jessica A. Eigeland for review and approval to ensure semantic equivalence.

- **Pilot Testing:**

A pilot study was conducted with 10 participants attending the Hacettepe University Family Medicine Clinics to evaluate item clarity and cultural appropriateness. Feedback was used to finalize the Turkish version.

- **Main Data Collection:**

The finalized scale was administered to the target sample. Participants were informed about the study objectives, and written informed consent was obtained. Data were collected through self-administered questionnaires.

### Statistical analysis

All analyses were performed using IBM SPSS Statistics 29.0 and JASP 0.18.3.Normality was assessed with the Kolmogorov–Smirnov test, which indicated non-normal distribution of total scale scores . Accordingly, Mann–Whitney *U* and Kruskal–Wallis *H* tests were used for group comparisons, with Bonferroni-adjusted post hoc tests where appropriate. Continuous variables are reported as median (min–max) and categorical variables as frequency (percentage). Construct validity was examined by confirmatory factor analysis (CFA) in JASP using the diagonally weighted least squares (DWLS) estimator suitable for ordinal, non-normally distributed data. Model fit was evaluated with *χ* ^2^, comparative fit index (CFI), Tucker–Lewis index (TLI), root mean square error of approximation (RMSEA) (90% CI), standardized root mean square residual (SRMR), goodness-of-fit index (GFI), McDonald fit index (MFI), Expected cross-validation index (ECVI), and Hoelter’s *N*, while sampling adequacy and factorability were checked using the Kaiser–Meyer–Olkin (KMO) measure and Bartlett’s test of sphericity. Internal consistency was assessed by Cronbach’s α, McDonald’s *ω*, and corrected item–total correlations. All tests were two-tailed, and *p* < 0.05 was considered statistically significant.

### Ethical issues

Ethical approval for the study was granted by the Hacettepe University Health Sciences Research Ethics Committee on Apr 8th, 2025 (Approval No: 2025/08-15). Participation was entirely voluntary, and all ethical principles including confidentiality, informed consent, and the right to withdraw were strictly adhered to.

## Results

### Participant characteristics

A total of 254 participants were included in the analysis (129 women and 125 men).The overall median total scale score was 83 (range = 22–110). Table 1 summarizes the group comparisons of total scale scores across sociodemographic and health-service–related variables.

**Table 1.** Group comparisons of total scale score by sociodemographic characteristics

Variable	Category (*n*)	Median (Min–Max)	Test statistic (U/H)	df	*p*	Significant pairwise comparisons (Bonferroni-adjusted)
Gender	Female (129)	86 (22–110)	7402.0	–	0.258	–
	Male (125)	81 (22–110)	–	–	–	–
Education level	Literate (25)	87 (22–110)	3.09	3	0.379	–
	Primary (87)	85 (22–110)	–	–	–	–
	High school (61)	85 (43–110)	–	–	–	–
	University+ (81)	78 (22–110)	–	–	–	–
Marital status	Single (34)	88 (43–110)	2409.0	–	< 0.001	Married vs. single (*p* < 0.001)
	Married (220)	81 (22–110)	–	–	–	–
Employment status	Unemployed (63)	83 (22–110)	28.80	3	< 0.001	Employed vs. retired (*p* < 0.001); Unemployed vs. retired (*p* = 0.019)
	Employed (106)	76 (22–110)	–	–	–	–
	Student (2)	91 (88–94)	–	–	–	–
	Retired (83)	88 (22–110)	–	–	–	–
Socioeconomic/cultural status	Low (20)	88 (22–110)	0.22	2	0.897	–
	Medium (189)	83 (22–110)	–	–	–	–
	High (45)	81 (60–110)	–	–	–	–
Frequency of visiting family physician	>1/month (49)	81 (64–110)	7.18	4	0.127	–
	Every 3 months (143)	81 (22–110)	–	–	–	–
	A few times/year (10)	87 (22–102)	–	–	–	–
	Only when needed (49)	86 (22–110)	–	–	–	–
	Never (3)	110 (80–110)	–	–	–	–
Type of most frequently visited health institution	Family health centre (177)	81 (22–110)	11.28	3	0.010	Public hospital vs. private hospital/clinic (*p* = 0.012); family health centre vs. private hospital/clinic (*p* = 0.033)
	Public hospital (52)	78 (22–110)	–	–	–	–
	University hospital (15)	88 (60–110)	–	–	–	–
	Private hospital/clinic (10)	89 (86–110)	–	–	–	–

### Group comparisons of total scale score

Gender was not associated with significant differences in total scale scores (*U* = 7402.0, *p* = 0.258).Similarly, no significant differences were observed across education levels (*H* = 3.09, df = 3, *p* = 0.379), socioeconomic/cultural status (*H* = 0.22, df = 2, *p* = 0.897), or frequency of visiting a family physician (*H* = 7.18, df = 4, *p* = 0.127).

By contrast, marital status showed a significant association with total scale scores (*U* = 2409.0, *p* < 0.001). Participants who were married had lower median scores (81, range = 22–110) than single participants (88, range = 43–110), and post hoc pairwise comparison confirmed this difference (Bonferroni-adjusted *p* < 0.001).

Employment status was also significantly related to total scale scores (*H* = 28.80, df = 3, *p* < 0.001). Post hoc tests revealed that retired individuals (median = 88, range = 22–110) scored significantly higher than both employed participants (median = 76, range = 22–110; *p* < 0.001) and unemployed participants (median = 83, range = 22–110; *p* = 0.019).

Finally, the type of most frequently visited health institution was associated with total scale scores (*H* = 11.28, df = 3, *p* = 0.010). Participants who most often used private hospitals or clinics (median = 89, range = 86–110) had significantly higher scores than those visiting public hospitals (median = 78, range = 22–110; *p* = 0.012) or family health centres (median = 81, range = 22–110; *p* = 0.033).No significant difference emerged between university hospitals and other institutions.

### Construct validity

CFA supported the proposed one-factor structure of the scale (Table 2).

**Table 2.** Construct validity of the scale

Analysis/fit index	Result
Chi-square (*χ* ^2^, df, p)	59.847 (df = 209), *p* = 1.000
Baseline model *χ* ^2^ (df = 231)	10 513.747
Comparative fit index (CFI)	1.000
Tucker–Lewis index (TLI)	1.016
Root mean square error of approximation (RMSEA)	0.000 (90% CI = 0.000–0.000), *p* = 1.000
Standardized root mean square residual (SRMR)	0.048
Goodness-of-fit index (GFI)	0.995
McDonald fit index (MFI)	1.343
Expected cross-validation index (ECVI)	0.584
Hoelter’s critical *N* (*α* = .05/.01)	1031.3/1097.9
Kaiser–Meyer–Olkin (KMO) overall measure	0.970
Bartlett’s test of sphericity	*χ* ^2^(231) = 5934.429, *p* < 0.001
Standardized factor loadings (22 items)	0.636 – 0.814 (all *p* < 0.001)

*Note:* All fit indices indicate an excellent fit for the one-factor model (e.g., CFI = 1.000; RMSEA = 0.000), while the KMO measure shows superb sampling adequacy (0.970) and Bartlett’s test confirms factorability (*p* < .001). All standardized factor loadings are high (0.636–0.814), providing strong evidence of construct validity.

The model demonstrated excellent overall fit: *χ* ^2^(209) = 59.847, *p* = 1.000; CFI = 1.000; TLI = 1.016; RMSEA = 0.000 (90% CI = 0.000–0.000, *p* = 1.000); SRMR = 0.048; GFI = 0.995; MFI = 1.343; and ECVI = 0.584.

Hoelter’s critical N was well above recommended thresholds (*α* = .05: 1031.3; *α* = .01: 1097.9), indicating model stability across large samples.

The KMO measure was 0.970, well above the .90 benchmark for “superb” adequacy, and Bartlett’s test of sphericity was significant (*χ* ^2^[231] = 5934.429, *p* < 0.001), confirming that correlations among items were sufficiently large for factor analysis.

All standardized factor loadings were strong and statistically significant, ranging from 0.636 to 0.814 (all *p* < 0.001), indicating that each item contributed meaningfully to the latent construct.

**Figure 1.**
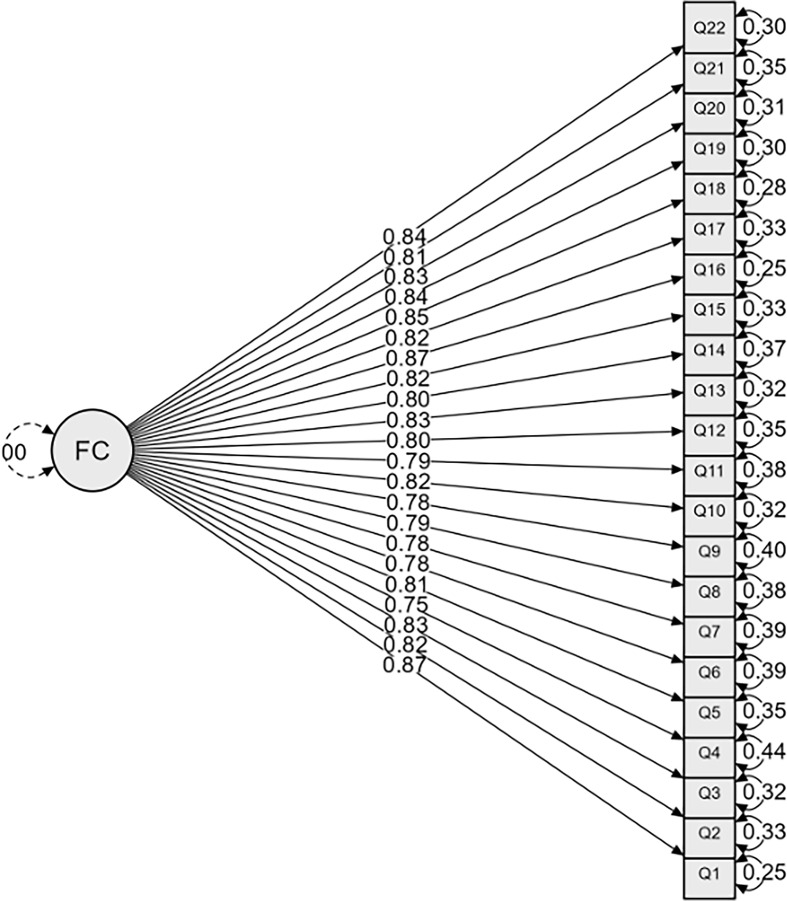
Confirmatory factor analysis path diagram. Standardized factor loadings (0.75–0.87) are shown on the single-headed arrows from the latent factor (FC) to the observed items (Q1–Q22). Residual variances (0.25–0.44) appear beside each item.

The CFA model is illustrated in Figure 1.

Standardized factor loadings (0.75–0.87) are shown on the single-headed arrows from the latent factor (FC) to the observed items (Q1–Q22). Residual variances (0.25–0.44) appear beside each item.

All 22 observed variables (Q1–Q22) loaded on a single latent factor (FC) with high standardized loadings ranging from 0.75 to 0.87 (all *p* < 0.001), supporting the unidimensional structure of the scale.

Error variances were low (0.25–0.44), indicating limited unexplained variance at the item level.

### Reliability

The scale demonstrated excellent internal consistency (Table 3).

**Table 3.** Scale reliability

Statistic	Value
Cronbach’s *α*	0.977
McDonald’s *ω*	0.976
Corrected item–total correlations (item–rest r)	0.74–0.86

*Note:* All corrected item–total correlations were well above the recommended minimum of 0.30, indicating that every item contributed strongly to the internal consistency of the scale.

Cronbach’s *α* was 0.977 and McDonald’s *ω* was 0.976, both far exceeding the commonly accepted threshold of 0.70 for good reliability.

Corrected item–total correlations ranged from 0.74 to 0.86, well above the recommended minimum of 0.30, showing that each item contributed strongly and consistently to the overall construct.

These findings indicate that the scale provides highly reliable measurement across all items.

## Discussion

The present study evaluated the psychometric properties of the Turkish version of the Chronic Conditions Patient–Physician Relationship Scale (CC-PPR) and confirmed its excellent reliability and validity among patients with chronic diseases. The results demonstrate that the Turkish adaptation of the CC-PPR is a robust, unidimensional, and highly consistent instrument for assessing the quality of doctor–patient relationships in primary care and chronic disease management settings.

CFA provided strong empirical support for the proposed one-factor model, consistent with the original validation study by Eigeland et al. (2025). The fit indices in the current sample (CFI = 1.000, TLI = 1.016, RMSEA = 0.000, SRMR = 0.048) exceeded conventional cut-off criteria and even surpassed those of the original English version (CFI = 0.95, TLI = 0.95, RMSEA = 0.07). Together with an excellent KMO value (0.970) and significant Bartlett’s test, these findings confirm that the Turkish CC-PPR captures a coherent latent construct representing patient-perceived physician relationship quality. The high standardized loadings (0.63–0.81) indicate that each item contributes meaningfully to the overall construct and that the conceptual structure of the scale remains stable across cultural contexts (Karagöz and Kösterelioğlu, 2008).

The Turkish version demonstrated excellent internal consistency (Cronbach’s *α* = 0.977; McDonald’s *ω* = 0.976), closely mirroring the reliability of the original instrument (*α* = 0.98). All corrected item–total correlations were above 0.70, confirming that the items function homogeneously and measure a single construct. These results reinforce the robustness and internal coherence of the CC-PPR and verify its suitability for both clinical and research applications. The very high reliability also supports its use in longitudinal and intervention studies evaluating communication and relationship-building skills among physicians.

Exploration of group differences revealed that perceived physician–patient relationship quality was not influenced by gender, educational attainment, socioeconomic status, or frequency of family-physician visits. This suggests that, within the Turkish context, relational quality is perceived similarly across demographic subgroups, emphasizing the universal and evolving nature of the doctor–patient relationship, as previously described by Kaba and Sooriakumaran (2007). They noted that while the relationship has shifted from a paternalistic to a more partnership-based model, fundamental elements such as empathy, trust, and mutual respect remain stable across cultural and demographic contexts (Kaba and Sooriakumaran, 2007).

However, significant differences emerged for marital and employment status as well as type of health institution. Single and retired participants reported higher relationship scores than married or actively employed individuals, possibly reflecting greater available time, more frequent physician interactions, or differing relational expectations. This finding may also align with interpersonal perception theories proposed by Kenny *et al*. (2010), who demonstrated that effective doctor–patient communication is shaped by mutual perception accuracy – that is, how accurately physicians and patients understand each other’s expectations and emotions – which may vary according to situational and contextual factors (Kenny *et al*., 2010).

Participants who most often visited private hospitals or clinics scored higher than those visiting public hospitals or family health centres, suggesting that institutional environment, continuity, and perceived accessibility may influence the quality of relational experiences. These contextual findings extend the existing literature and highlight potential structural determinants of relational satisfaction in chronic-care settings. As shown in a population-based study by Hajek *et al*. (2016), patients reporting a more positive family physician–patient relationship tend to use healthcare services less frequently (Hajek *et al*., 2016). Future studies using the Turkish CC-PPR may explore similar associations between relational quality and healthcare utilization patterns in Türkiye.

The psychometric equivalence between this adaptation and the original study confirms the cross-cultural generalizability of the CC-PPR (Eigeland *et al*., 2025). Both studies yielded a one-factor solution, excellent reliability, and strong construct validity, demonstrating that the dimensions underlying the physician–patient relationship in chronic disease management are conceptually universal.

The findings of this study are consistent with prior validations of doctor–patient relationship instruments, such as the Patient–Doctor Relationship Questionnaire (PDRQ-9), which has been extensively examined across various cultural contexts. Porcerelli *et al*. (2014) confirmed the strong internal consistency (Cronbach’s *α* = 0.94) and unidimensional structure of the PDRQ-9 in primary care, similar to the results obtained for the Turkish CC-PPR (*α* = 0.977) (Porcerelli *et al*., 2014). Both instruments emphasize patient-perceived relational quality, focusing on accessibility, trust, empathy, and the physician’s understanding of patient needs. However, the CC-PPR expands this conceptualization by incorporating items derived from patients with chronic conditions, thereby addressing the continuity and long-term collaboration aspects that are not fully captured by shorter measures such as the PDRQ-9.

The slightly higher fit indices observed in this study may reflect the high contextual relevance of long-term, trust-based relationships in Turkish family medicine practice, where continuity of care and personal familiarity are emphasized. Given that the quality of physician–patient relationships influences healthcare-seeking behaviour, as evidenced by Hajek et al. (2016), the validated Turkish CC-PPR provides an important tool for family physicians to monitor and enhance relational continuity, potentially improving both patient satisfaction and system efficiency (Hajek *et al*., 2016).

The validated Turkish CC-PPR offers an empirically supported instrument to evaluate and enhance the relational and communication competencies of family physicians and other primary-care providers managing chronic diseases. Its use can support patient-centred quality improvement initiatives, postgraduate training, and research on determinants of adherence, satisfaction, and continuity of care. Because the CC-PPR items reflect observable physician behaviours, the scale can also guide feedback-based educational interventions, facilitating reflection on communication style, empathy, and follow-up practices.

### Limitations

Several limitations should be acknowledged. The sample was drawn from a single university-based setting, which may limit generalizability to other regions or healthcare systems. Convergent validity could not be assessed using external scales such as the Working Alliance Inventory or CARE Measure due to survey length constraints.

## Conclusion

In summary, the Turkish version of the Chronic Conditions Physician–Patient Relationship Scale exhibits excellent psychometric properties, replicating the unidimensional structure, internal consistency, and construct validity of the original instrument. These results establish the CC-PPR as a culturally adaptable, patient-informed, and psychometrically sound measure of the doctor–patient relationship in chronic disease management. Its application in Turkish primary care will contribute to promoting patient-centred communication, improving adherence, and strengthening the relational dimension of chronic-care delivery.
